# Supplementary material for: Generalized Approach towards Secretion-Based Protein Production via Neutralization of Secretion-Preventing Cationic Substrate Residues
Source: Int J Mol Sci. 2022 Jun 15;23(12):6700. doi: 10.3390/ijms23126700 (PMC9223453; doi:10.3390/ijms23126700)
Supplement: Supplementary file 1 [file ijms-23-06700-s001.zip › Supplementary ver 5.2_IJMS_Byun.pdf]

Generalized Approach towards Secretion-Based Protein Production via Neutralization of Secretion-Preventing Cationic Substrate Residues (Supplementary Text S1, Table S1, and Table S2)

Hyunjong Byun [1], Jiyeon Park [2], Benedict U. Fabia [2], Joshua Bingwa [2], Mihn Hieu Nguyen [2], Haeshin Lee [3], and Jung Hoon Ahn [1,2*]

1 Department of Biological Sciences, Korea Advanced Institute of Science and Technology (KAIST), Daejeon 34141, Republic of Korea
2 Department of Chemistry and Biology, Korea Science Academy of Korea Advanced Institute of Science and Technology, Busan 47162, Republic of Korea
3 Department of Chemistry, Korea Advanced Institute of Science and Technology, Daejeon 34141, Republic of Korea
* Correspondence: (J.H.A.) hoony@kaist.ac.kr; Tel.: (+82)10-8826-6109; (H.L.) haeshin@kaist.ac.kr; Tel.: (+82)10-8974-5141

Text S1. Sequences of proteins in FASTA format

Color code for enzyme sites and polypeptide features

Multiple cloing site (MCS):
XbaI: tctaga, SR NdeI: catatg, HM KpnI: ggtacc, GT
NheI: gctagc, AS SacI: gagctc, EL

Factor Xa cleavage site: IEGR

LARD3 signal peptide:
GSDGNDLIQGGKGADFIEGGKGNDTIRDNSGHNTFLFSGHFGQDRIIGYQPTDRLVFQGADGSTDLRDHAKAVG
ADTVLSFGADSVTLVGVGLGGLWSEGVLIS

Polyhistidine tag (His-tag): HHHHHH

>TliA, wild type (as a reference)
MGVFDYKNLGTEASKTLFADATAITLYTYHNLDNGFAVGYQQHGLGLGLPATLVGALLGSTDSQGVIPGIPWNP
DSEKAALDAVHAAGWTPISASALGYGGKVDARGTFFGEKAGYTTAQAEVLGKYDDAGKLLEIGIGFRGTSGPRE
SLITDSIGDLVSDLLAALGPKDYAKNYAGEAFGGLLKTVADYAGAHGLSGKDVLVSGHSLGGLAVNSMADLSTS
KWAGFYKDANYLAYASPTQSAGDKVLNIGYENDPVFRALDGSTFNLSSLGVHDKAHESTTDNIVSFNDHYASTL
WNVLPFSIANLSTWVSHLPSAYGDGMTRVLESGFYEQMTRDSTIIVANLSDPARANTWVQDLNRNAEPHTGNTF
IIGSDGNDLIQGGKGADFIEGGKGNDTIRDNSGHNTFLFSGHFGQDRIIGYQPTDRLVFQGADGSTDLRDHAKA
VGADTVLSFGADSVTLVGVGLGGLWSEGVLIS

>TliA, expressed in pDART plasmid (this is used for computational analysis) [14]
MSRMGVFDYKNLGTEASKTLFADATAITLYTYHNLDNGFAVGYQQHGLGLGLPATLVGALLGSTDSQGVIPGIP
WNPDSEKAALDAVHAAGWTPISASALGYGGKVDARGTFFGEKAGYTTAQAEVLGKYDDAGKLLEIGIGFRGTSG
PRESLITDSIGDLVSDLLAALGPKDYAKNYAGEAFGGLLKTVADYAGAHGLSGKDVLVSGHSLGGLAVNSMADL
STSKWAGFYKDANYLAYASPTQSAGDKVLNIGYENDPVFRALDGSTFNLSSLGVHDKAHESTTDNIVSFNDHYA
STLWNVLPFSIANLSTWVSHLPSAYGDGMTRVLESGFYEQMTRDSTIIVANLSDPARANTWVQDLNRNAEPHTG
NTFIIGSDGNDLIQGGKGADFIEGGKGNDTIRDNSGHNTFLFSGHFGQDRIIGYQPTDRLVFQGADGSTDLRDH
AKAVGADTVLSFGADSVTLVGVGLGGLWSEGVLISELIEGRGSDGNDLIQGGKGADFIEGGKGNDTIRDNSGHN

TFLFSGHFGQDRIIGYQPTDRLVFQGADGSTDLRDHAKAVGADTVLSFGADSVTLVGVGLGGLWSEGVLIS

>NKC-TliA: NKC is marked cyan. [14]
MSRHMGTAPKAMKLLKKLLKLQKKGIGSMGVFDYKNLGTEASKTLFADATAITLYTYHNLDNGFAVGYQQHGLG
LGLPATLVGALLGSTDSQGVIPGIPWNPDSEKAALDAVHAAGWTPISASALGYGGKVDARGTFFGEKAGYTTAQ
AEVLGKYDDAGKLLEIGIGFRGTSGPRESLITDSIGDLVSDLLAALGPKDYAKNYAGEAFGGLLKTVADYAGAH
GLSGKDVLVSGHSLGGLAVNSMADLSTSKWAGFYKDANYLAYASPTQSAGDKVLNIGYENDPVFRALDGSTFNL
SSLGVHDKAHESTTDNIVSFNDHYASTLWNVLPFSIANLSTWVSHLPSAYGDGMTRVLESGFYEQMTRDSTIIV
ANLSDPARANTWVQDLNRNAEPHTGNTFIIGSDGNDLIQGGKGADFIEGGKGNDTIRDNSGHNTFLFSGHFGQD
RIIGYQPTDRLVFQGADGSTDLRDHAKAVGADTVLSFGADSVTLVGVGLGGLWSEGVLISELIEGRGSDGNDLI
QGGKGADFIEGGKGNDTIRDNSGHNTFLFSGHFGQDRIIGYQPTDRLVFQGADGSTDLRDHAKAVGADTVLSFG
ADSVTLVGVGLGGLWSEGVLIS

>CTP-TliA: CTP is marked cyan. [14]
MSRMRGSHHHHHHGMASMTGGQQMGRDLYDDDDKDRWGSMYGRRARRRRRRSMAGTGGMGVFDYKNLGTEASKT
LFADATAITLYTYHNLDNGFAVGYQQHGLGLGLPATLVGALLGSTDSQGVIPGIPWNPDSEKAALDAVHAAGWT
PISASALGYGGKVDARGTFFGEKAGYTTAQAEVLGKYDDAGKLLEIGIGFRGTSGPRESLITDSIGDLVSDLLA
ALGPKDYAKNYAGEAFGGLLKTVADYAGAHGLSGKDVLVSGHSLGGLAVNSMADLSTSKWAGFYKDANYLAYAS
PTQSAGDKVLNIGYENDPVFRALDGSTFNLSSLGVHDKAHESTTDNIVSFNDHYASTLWNVLPFSIANLSTWVS
HLPSAYGDGMTRVLESGFYEQMTRDSTIIVANLSDPARANTWVQDLNRNAEPHTGNTFIIGSDGNDLIQGGKGA
DFIEGGKGNDTIRDNSGHNTFLFSGHFGQDRIIGYQPTDRLVFQGADGSTDLRDHAKAVGADTVLSFGADSVTL
VGVGLGGLWSEGVLISELIEGRGSDGNDLIQGGKGADFIEGGKGNDTIRDNSGHNTFLFSGHFGQDRIIGYQPT
DRLVFQGADGSTDLRDHAKAVGADTVLSFGADSVTLVGVGLGGLWSEGVLIS

>Glutathione S-transferase, wild type (GST) [15]
MSRHHHHHHGGPPYTITYFPVRGRCEAMRMLLADQDQSWKEEVVTMETWPPLKPSCLFRQLPKFQDGDLTLYQS
NAILRHLGRSFGLYGKDQKEAALVDMVNDGVEDLRCKYATLIYTNYEAGKEKYVKELPEHLKPFETLLSQNQGG
QAFVVGSQISFADYNLLDLLRIHQVLNPSCLDAFPLLSAYVARLSARPKIKAFLASPEHVNRPINGNGKQELIE
GRGSDGNDLIQGGKGADFIEGGKGNDTIRDNSGHNTFLFSGHFGQDRIIGYQPTDRLVFQGADGSTDLRDHAKA
VGADTVLSFGADSVTLVGVGLGGLWSEGVLIS

>Glutathione S-transferase, negatively supercharged (GST(−20)) [15]
MSRHHHHHHGGPPYTITYFPVRGRCEAMRMLLADQDQSWEEEVVTMETWPPLKPSCLFRQLPKFQDGDLTLYQS
NAILRHLGRSFGLYGEDEEEAALVDMVNDGVEDLRCKYATLIYTDYEAGKEEYVEELPEHLKPFETLLSENEGG
EAFVVGSEISFADYNLLDLLRIHQVLNPSCLDAFPLLSAYVARLSARPEIEAFLASPEHVDRPINGNGKQELIE
GRGSDGNDLIQGGKGADFIEGGKGNDTIRDNSGHNTFLFSGHFGQDRIIGYQPTDRLVFQGADGSTDLRDHAKA
VGADTVLSFGADSVTLVGVGLGGLWSEGVLIS

>Glutathione S-transferase, positively supercharged (GST(+19)) – designed by us
MSRHHHHHHGGPPYTITYFPVRGRCEAMRMLLADQKQSWKEEVVTMKTWPPLKPSCLFRQLPKFQDGKLTLYQS
NAILRHLGRSFGLYGKDQKEAALVDMVNDGVEDLRCKYATLIYTNYKAGKQKYVKELPKHLKPFETLLSKNKGG
KAFVVGSKISFADYNLLDLLRIHQVLNPSCLKAFPLLSAYVARLSARPKIKAFLASPEHVKRPINGNGKQELIE
GRGSDGNDLIQGGKGADFIEGGKGNDTIRDNSGHNTFLFSGHFGQDRIIGYQPTDRLVFQGADGSTDLRDHAKA
VGADTVLSFGADSVTLVGVGLGGLWSEGVLIS

>Streptavidin, wild-type (SAv) [15]
MSRHHHHHHGGAEAGITGTWYNQLGSTFIVTAGADGALTGTYESAVGNAESRYVLTGRYDSAPATDGSGTALGW
TVAWKNNYRNAHSATTWSGQYVGGAEARINTQWLLTSGTTEANAWKSTLVGHDTFTKVKPSAASELIEGRGSDG
NDLIQGGKGADFIEGGKGNDTIRDNSGHNTFLFSGHFGQDRIIGYQPTDRLVFQGADGSTDLRDHAKAVGADTV
LSFGADSVTLVGVGLGGLWSEGVLIS

>Streptavidin, negatively supercharged (SAv(−10)) [15]
MSRHHHHHHGGAEAGITGTWYNQLGSTFIVTAGADGALTGTYESAVGDAESEYVLTGRYDSAPATDGSGTALGW
TVAWKNDYENAHSATTWSGQYVGGAEARINTQWLLTSGTTEADAWKSTLVGHDTFTKVEPSAASELIEGRGSDG
NDLIQGGKGADFIEGGKGNDTIRDNSGHNTFLFSGHFGQDRIIGYQPTDRLVFQGADGSTDLRDHAKAVGADTV
LSFGADSVTLVGVGLGGLWSEGVLIS

>Streptavidin, positively supercharged (SAv(+13)) [15]
MSRHHHHHHGGAKAGITGTWYNQLGSTFIVTAGAKGALTGTYESAVGNAKSRYVLTGRYDSAPATKGSGTALGW
TVAWKNKYRNAHSATTWSGQYVGGAKARINTQWLLTSGTTKAKAWKSTLVGHDTFTKVKPSAASELIEGRGSDG
NDLIQGGKGADFIEGGKGNDTIRDNSGHNTFLFSGHFGQDRIIGYQPTDRLVFQGADGSTDLRDHAKAVGADTV
LSFGADSVTLVGVGLGGLWSEGVLIS

>Cutinase (Cuti) [14]
MSRHHHHHHAPTSNPAQELEARQLGRTTRDDLINGNSASCADVIFIYARGSTETGNLGTLGPSIASNLESAFGK
DGVWIQGVGGAYRATLGDNALPRGTSSAAIREMLGLFQQANTKCPDATLIAGGYSQGAALAAASIEDLDSAIRD
KIAGTVLFGYTKNLQNRGRIPNYPADRTKVFCNTGDLVCTGSLIVAAPHLAYGPDARGPAPEFLIEKVRAVRGS
ALEELIEGRGSDGNDLIQGGKGADFIEGGKGNDTIRDNSGHNTFLFSGHFGQDRIIGYQPTDRLVFQGADGSTD
LRDHAKAVGADTVLSFGADSVTLVGVGLGGLWSEGVLIS

>Cutinase, negatively supercharged (Cuti(−))
MSRHHHHHHAPTSNPAQELEARQLGETTRDDLIDGDSASCADVIFIYARGSTETGNLGTLGPSIASNLESAFGE
DGVWIQGVGGAYEATLGDNALPRGTSSAAIEEMLGLFQQANTKCPDATLIAGGYSQGAALAAASIEDLDSAIRD
KIAGTVLFGYTKNLENEGRIPNYPADRTKVFCNTGDLVCTGSLIVAAPHLAYGPDAEGPAPEFLIEKVRAVRGS
ALEELIEGRGSDGNDLIQGGKGADFIEGGKGNDTIRDNSGHNTFLFSGHFGQDRIIGYQPTDRLVFQGADGSTD
LRDHAKAVGADTVLSFGADSVTLVGVGLGGLWSEGVLIS

>Chitinase (Chi) [14]
MSRHHHHHHANSPKQSQKIVGYFPSWGVYGRNYQVADIDASKLTHLNYAFADICWNGKHGNPSTHPDNPNKQTW
NCKESGVPLQNKEVPNGTLVLGEPWADVTKSYPGSGTTWEDCDKYARCGNFGELKRLKAKYPHLKTIISVGGWT
WSNRFSDMAADEKTRKVFAESTVAFLRAYGFDGVDLDWEYPGVETIPGGSYRPEDKQNFTLLLQDVRNALNKAG
AEDGKQYLLTIASGASRRYADHTELKKISQILDWINIMTYDFHGGWEATSNHNAALYKDPNDPAANTNFYVDGA
INVYTNEGVPVDKLVLGVPFYGRGWKSCGKENNGQYQPCKPGSDGKLASKGTWDDYSTGDTGVYDYGDLAANYV
NKNGFVRYWNDTAKVPYLYNATTGTFISYDDNESMKYKTDSIKTKGLSGAMFWELSGDCRTSPKYSCSGPKLLD
TLVKELLGGPINQKDTEPPTNVKNIVVTNKNSNSVQLNWTASTDNVGVTEYEITAGEEKWSTTTNSITIKNLKP
NTEYKFSIIAKDAAGNKSQPTALTVKTDEANMTPPDGNGTATFSVTSNWGSGYNFSIIIKNNGTNPIKNWKLEF
DYSGNLTQVWDSKISSKTNNHYVITNAGWNGEIPPGGSITIGGAGTGNPAELLNAVISENELIEGRGSDGNDLI
QGGKGADFIEGGKGNDTIRDNSGHNTFLFSGHFGQDRIIGYQPTDRLVFQGADGSTDLRDHAKAVGADTVLSFG
ADSVTLVGVGLGGLWSEGVLIS

>Chitinase, negatively supercharged (Chi(−))
MSRHHHHHHANSPEQSQKIVGYFPSWGVYGRDYQVADIDASKLTHLNYAFADICWNGEHGNPSTHPDNPNEQTW
NCEESGVPLQNKEVPNGTLVLGEPWADVTKSYPGSGTTWEDCDEYARCGNFGELKRLKAEYPHLKTIISVGGWT
WSNRFSDMAADEETREVFAESTVAFLEAYGFDGVDLDWEYPGVETIPGGSYRPEDKQNFTLLLQDVRNALNEAG
AEDGEQYLLTIASGASRRYADHTELKKISQILDWINIMTYDFHGGWEATSNHNAALYKDPNDPAANTNFYVDGA
INVYTNEGVPVDKLVLGVPFYGRGWKSCGKENDGQYQPCKPGSDGKLASEGTWDDYSTGDTGVYDYGDLAANYV
DEDGFVRYWNDTAKVPYLYNATTGTFISYDDNESMKYKTDSIKTKGLSGAMFWELSGDCRTSPEYSCSGPKLLD
TLVKELLGGPIDEKDTEPPTNVKNIVVTNKNSNSVQLNWTASTDNVGVTEYEITAGEEKWSTTTNSITIKNLKP
NTEYEFSIIAKDAAGNKSQPTALTVKTDEADMTPPDGNGTATFSVTSNWGSGYNFSIIIEDDGTNPIKNWKLEF
DYSGNLTQVWDSKISSETNNHYVITNAGWNGEIPPGGSITIGGAGTGDPAELLNAVISEDELIEGRGSDGNDLI
QGGKGADFIEGGKGNDTIRDNSGHNTFLFSGHFGQDRIIGYQPTDRLVFQGADGSTDLRDHAKAVGADTVLSFG

ADSVTLVGVGLGGLWSEGVLIS

>MelC2 tyrosinase (MelC2)
MSSMTVRKNQASLTAEEKRRFVAALLELKRTGRYDAFVTTHNAFILGDTDNGERTGHRSPSFLPWHRRFLLEFE
RALQSVDASVALPYWDWSADRSTRSSLWAPDFLGGTGRSRDGQVMDGPFAASAGNWPINVRVDGRTFLRRALGA
GVSELPTRAEVDSVLAMATYDMAPWNSGSDGFRNHLEGWRGVNLHNRVHVWVGGQMATGVSPNDPVFWLHHAYI
DKLWAEWQRRHPSSPYLPGGGTPNVVDLNETMKPWNDTTPAALLDHTRHYTFDVASELIEGRGSDGNDLIQGGK
GADFIEGGKGNDTIRDNSGHNTFLFSGHFGQDRIIGYQPTDRLVFQGADGSTDLRDHAKAVGADTVLSFGADSV
TLVGVGLGGLWSEGVLIS

>MelC2 tyrosinase, negatively supercharged extensively (MelC2(−40))
MSRHHHHHHTVRENQASLTAEEKEEFVAALLELKETGEYDAFVTTHNAFILGDTDDGERTGHRSPSFLPWHRRF
LLEFEEALQSVDASVALPYWDWSADRSTESSLWAPDFLGGTGESEDGQVMDGPFAASAGDWPIDVEVDGETFLR
RALGAGVSELPTEAEVDSVLAMATYDMAPWNSGSDGFRNHLEGWEGVNLHNRVHVWVGGQMATGVSPNDPVFWL
HHAYIDKLWAEWQEEHPSSPYLPGGGTPDVVDLDETMEPWDDTTPAALLDHTEHYTFDVELIEGRGSDGNDLIQ
GGKGADFIEGGKGNDTIRDNSGHNTFLFSGHFGQDRIIGYQPTDRLVFQGADGSTDLRDHAKAVGADTVLSFGA
DSVTLVGVGLGGLWSEGVLIS

>MelC2 tyrosinase, negatively supercharged defensively (MelC2(−20))
MSRHHHHHHTVRKNQASLTAEEKERFVAALLELKETGRYDAFVTTHNAFILGDTDDGERTGHRSPSFLPWHRRF
LLEFEEALQSVDASVALPYWDWSADRSTESSLWAPDFLGGTGESEDGQVMDGPFAASAGNWPINVRVDGRTFLR
RALGAGVSELPTEAEVDSVLAMATYDMAPWNSGSDGFRNHLEGWRGVNLHNRVHVWVGGQMATGVSPNDPVFWL
HHAYIDKLWAEWQERHPSSPYLPGGGTPDVVDLDETMKPWDDTTPAALLDHTEHYTFDVELIEGRGSDGNDLIQ
GGKGADFIEGGKGNDTIRDNSGHNTFLFSGHFGQDRIIGYQPTDRLVFQGADGSTDLRDHAKAVGADTVLSFGA
DSVTLVGVGLGGLWSEGVLIS

>MelC2 tyrosinase, selectively superneutralized (MelC2(Q))
MSRHHHHHHTVRQNQASLTAEEKRQFVAALLELKQTGRYDAFVTTHNAFILGDTDNGERTGHRSPSFLPWHRRF
LLEFEQALQSVDASVALPYWDWSADQSTQSSLWAPDFLGGTGRSQDGQVMDGPFAASAGNWPINVQVDGQTFLR
RALGAGVSELPTQAEVDSVLAMATYDMAPWNSGSDGFRNHLEGWQGVNLHNRVHVWVGGQMATGVSPNDPVFWL
HHAYIDKLWAEWQQQHPSSPYLPGGGTPNVVDLNETMQPWNDTTPAALLDHTEHYTFDVELIEGRGSDGNDLIQ
GGKGADFIEGGKGNDTIRDNSGHNTFLFSGHFGQDRIIGYQPTDRLVFQGADGSTDLRDHAKAVGADTVLSFGA
DSVTLVGVGLGGLWSEGVLIS

>M37 lipase (M37) [14]
MSRHMSYTKEQLMLAFSYMSYYGITHTGSAKKNAELILKKMKEALKTWKPFQEDDWEVVWGPAVYTMPFTIFND
AMMYVIQKKGAEGEYVIAIRGTNPVSISDWLFNDFMVSAMKKWPYASVEGRILKISESTSYGLKTLQKLKPKSH
IPGENKTILQFLNEKIGPEGKAKICVTGHSKGGALSSTLALWLKDIQGVKLSQNIDISTIPFAGPTAGNADFAD
YFDDCLGDQCTRIANSLDIVPYAWNTNSLKKLKSIYISEQASVKPLLYQRALIRAMIAETKGKKYKQIKAETPP
LEGNINPILIEYLVQAAYQHVVGYPELMGMMDDIPLTDIFEDAIAGLLLEHHHHHHGTASELIEGRGSDGNDLI
QGGKGADFIEGGKGNDTIRDNSGHNTFLFSGHFGQDRIIGYQPTDRLVFQGADGSTDLRDHAKAVGADTVLSFG
ADSVTLVGVGLGGLWSEGVLIS

>M37 lipase, negatively supercharged extensively (M37(−23))
MSRHHHHHHSYTKEQLMLAFSYMSYYGITHTGSAKENAELILEKMKEALETWEPFQEDDWEVVWGPAVYTMPFT
IFNDAMMYVIQKEGAEGEYVIAIRGTNPVSISDWLFNDFMVSAMKEWPYASVEGEILKISESTSYGLKTLQELK
PKSHIPGEDKTILEFLNEKIGPEGEAKICVTGHSKGGALSSTLALWLKDIQGVELSEDIDISTIPFAGPTAGNA
DFADYFDDCLGDQCTRIANSLDIVPYAWNTDSLEELKSIYISEEASVEPLLYQRALIEAMIAETEGKEYKQIKA
ETPPLEGDINPILIEYLVQAAYQHVVGYPELMGMMDDIPLTDIFEDAIAGLLELIEGRGSDGNDLIQGGKGADF
IEGGKGNDTIRDNSGHNTFLFSGHFGQDRIIGYQPTDRLVFQGADGSTDLRDHAKAVGADTVLSFGADSVTLVG

VGLGGLWSEGVLIS

>M37 lipase, negatively supercharged defensively (M37(−14))
MSRHHHHHHSYTKEQLMLAFSYMSYYGITHTGSAKKNAELILEKMKEALETWEPFQEDDWEVVWGPAVYTMPFT
IFNDAMMYVIQKEGAEGEYVIAIRGTNPVSISDWLFNDFMVSAMKKWPYASVEGRILKISESTSYGLKTLQELK
PKSHIPGEDKTILQFLNEKIGPEGEAKICVTGHSKGGALSSTLALWLKDIQGVELSQDIDISTIPFAGPTAGNA
DFADYFDDCLGDQCTRIANSLDIVPYAWNTNSLKKLKSIYISEQASVKPLLYQRALIRAMIAETKGKKYKQIKA
ETPPLEGNINPILIEYLVQAAYQHVVGYPELMGMMDDIPLTDIFEDAIAGLLELIEGRGSDGNDLIQGGKGADF
IEGGKGNDTIRDNSGHNTFLFSGHFGQDRIIGYQPTDRLVFQGADGSTDLRDHAKAVGADTVLSFGADSVTLVG
VGLGGLWSEGVLIS

>M37 lipase, selectively superneutralized (M37(Q))
MSRHHHHHHSYTKEQLMLAFSYMSYYGITHTGSAKQNAELILQKMKEALQTWKPFQEDDWEVVWGPAVYTMPFT
IFNDAMMYVIQKQGAEGEYVIAIRGTNPVSISDWLFNDFMVSAMQQWPYASVEGRILQISESTSYGLKTLQQLQ
PQSHIPGENQTILQFLNEKIGPEGQAQICVTGHSKGGALSSTLALWLKDIQGVQLSQNIDISTIPFAGPTAGNA
DFADYFDDCLGDQCTRIANSLDIVPYAWNTNSLQQLKSIYISEQASVKPLLYQQALIQAMIAETQGQQYKQIKA
ETPPLEGNINPILIEYLVQAAYQHVVGYPELMGMMDDIPLTDIFEDAIAGLLELIEGRGSDGNDLIQGGKGADF
IEGGKGNDTIRDNSGHNTFLFSGHFGQDRIIGYQPTDRLVFQGADGSTDLRDHAKAVGADTVLSFGADSVTLVG
VGLGGLWSEGVLIS

>M37 lipase, randomly mutated (design of M37(var))
MSRHHHHHHSYTKEQLMLAFSYMSYYGITHTGSAKeNAELILEKMKEALETWEPFQEDDWEVVWGPAVYTMPFT
IFNDAMMYVIQKEGAEGEYVIAIRGTNPVSISDWLFNDFMVSAMKeWPYASVEGeILKISESTSYGLKTLQELK
PKSHIPGEDKTILZFLNEKIGPEGEAKICVTGHSKGGALSSTLALWLKDIQGVELSZDIDISTIPFAGPTAGNA
DFADYFDDCLGDQCTRIANSLDIVPYAWNTBSLeeLKSIYISEZASVePLLYQRALIeAMIAETeGKeYKQIKA
ETPPLEGBINPILIEYLVQAAYQHVVGYPELMGMMDDIPLTDIFEDAIAGLLELIEGRGSDGNDLIQGGKGADF
IEGGKGNDTIRDNSGHNTFLFSGHFGQDRIIGYQPTDRLVFQGADGSTDLRDHAKAVGADTVLSFGADSVTLVG
VGLGGLWSEGVLIS
e indicates “Lys or Glu”, or “K or E”, which is encoded by codon RAG.
B indicates “Asn or Asp”, or “N or D”, which is encoded by codon RAC.
Z indicates “Gln or Glu”, or “Q or E”, which is encoded by codon SAG.

>M37 lipase, randomly mutated and activity-based screened (M37(var))
MSRHHHHHHSYTKEQLMLAFSYMSYYGITHTGSAKKNAELILEKMKEALETWEPFQEDDWEVVWGPAVYTMPFT
IFNDAMMYVIQKEGAEGEYVIAIRGTNPVSISDWLFNDFMVSAMKKWPYASVEGKVLKISESTSYGLKTLQELK
PKSHIPGEDKTILQFLNEKIGPEGEAKICVTGHSKGGALSSTLALWLKDIQGVELSEDIDISTIPFAGPTAGNA
DFADYFDDCLGDQCTRIANSLDIVPYAWNTNSLKELKSIYISEEASVKPLLYQRALIEAMIAETEGKEYKQIKA
ETPPLEGNINPILIEYLVQAAYQHVVGYPELMGMMDDIPLTDIFEDAIAGLLELIEGRGSDGNDLIQGGKGADF
IEGGKGNDTIRDNSGHNTFLFSGHFGQDRIIGYQPTDRLVFQGADGSTDLRDHAKAVGADTVLSFGADSVTLVG
VGLGGLWSEGVLIS

>Green fluorescent protein (GFP) [14]
MSRMSKGEELFTGVVPILVELDGDVNGHKFSVSGEGEGDATYGKLTLKFICTTGKLPVPWPTLVTTFSYGVQCF
SRYPDHMKRHDFFKSAMPEGYVQERTISFKDDGNYKTRAEVKFEGDTLVNRIELKGIDFKEDGNILGHKLEYNY
NSHNVYITADKQKNGIKANFKIRHNIEDGSVQLADHYQQNTPIGDGPVLLPDNHYLSTQSALSKDPNEKRDHMV
LLEFVTAAGITHGMDELIEGRGSDGNDLIQGGKGADFIEGGKGNDTIRDNSGHNTFLFSGHFGQDRIIGYQPTD
RLVFQGADGSTDLRDHAKAVGADTVLSFGADSVTLVGVGLGGLWSEGVLIS

>−30 Negatively supercharged GFP (GFP(−30)) [14, 15]
MSRMGHHHHHHGGASKGEELFDGVVPILVELDGDVNGHEFSVRGEGEGDATEGELTLKFICTTGELPVPWPTLV

TTLTYGVQCFSDYPDHMDQHDFFKSAMPEGYVQERTISFKDDGTYKTRAEVKFEGDTLVNRIELKGIDFKEDGN
ILGHKLEYNFNSHDVYITADKQENGIKAEFEIRHNVEDGSVQLADHYQQNTPIGDGPVLLPDDHYLSTESALSK
DPNEDRDHMVLLEFVTAAGIDHGMDELYKELIEGRGSDGNDLIQGGKGADFIEGGKGNDTIRDNSGHNTFLFSG
HFGQDRIIGYQPTDRLVFQGADGSTDLRDHAKAVGADTVLSFGADSVTLVGVGLGGLWSEGVLIS

>Transforming growth factor β (TGFβ)

MSRHHHHHHMSRALDTNYCFSSTEKNCCVRQLYIDFRKDLGWKWIHEPKGYHANFCLGPCPYIWSLDTQYSKVL
ALYNQHNPGASAAPCCVPQALEPLPIVYYVGRKPKVEQLSNMIVRSCKCSELIEGRGSDGNDLIQGGKGADFIE
GGKGNDTIRDNSGHNTFLFSGHFGQDRIIGYQPTDRLVFQGADGSTDLRDHAKAVGADTVLSFGADSVTLVGVG
LGGLWSEGVLIS

>Transforming growth factor β, LCD-based supercharged (TGFβ(−))

MSRHHHHHHMSRALDTNYCFSSTEENCCVRQLYIDFREDLGWKWIHEPKGYHANFCLGPCPYIWSLDTQYSKVL
ALYNQHNPGASAAPCCVPQALEPLPIVYYVGRDPKVEQLSNMIVDSCKCSELIEGRGSDGNDLIQGGKGADFIE
GGKGNDTIRDNSGHNTFLFSGHFGQDRIIGYQPTDRLVFQGADGSTDLRDHAKAVGADTVLSFGADSVTLVGVG
LGGLWSEGVLIS

>Tumor necrosis factor β (TNFβ)

MSRHHHHHHMLPGVGLTPSAAQTARQHPKMHLAHSTLKPAAHLIGDPSKQNSLLWRANTDRAFLQDGFSLSNNS
LLVPTSGIYFVYSQVVFSGKAYSPKATSSPLYLAHEVQLFSSQYPFHVPLLSSQKMVYPGLQEPWLHSMYHGAA
FQLTQGDQLSTHTDGIPHLVLSPSTVFFGAFALELIEGRGSDGNDLIQGGKGADFIEGGKGNDTIRDNSGHNTF
LFSGHFGQDRIIGYQPTDRLVFQGADGSTDLRDHAKAVGADTVLSFGADSVTLVGVGLGGLWSEGVLIS

>Tumor necrosis factor β, LCD-based supercharged ((TNFβ(−))

MSRHHHHHHMLPGVGLTPSAAQTAQQHPQMHLAHSTLKPAAHLIGDPSTQNSLLWRANTDRAFLQDGFSLSNNS
LLVPTSGIYFVYSQVVFSGEAYSPEATSSPLYLAHEVQLFSSQYPFHVPLLSSQKMVYPGLQEPWLHSMYHGAA
FQLTQGDQLSTHTDGIPHLVLSPSTVFFGAFALELIEGRGSDGNDLIQGGKGADFIEGGKGNDTIRDNSGHNTF
LFSGHFGQDRIIGYQPTDRLVFQGADGSTDLRDHAKAVGADTVLSFGADSVTLVGVGLGGLWSEGVLIS

>Fibroblast growth factor 1 (FGF1)

MSRHHHHHHMFNLPPGNYKKPKLLYCSNGGHFLRILPDGTVDGTRDRSDQHIQLQLSAESVGEVYIKSTETGQY
LAMDTDGLLYGSQTPNEECLFLERLEENHYNTYISKKHAEKNWFVGLKKNGSCKRGPRTHYGQKAILFLPLPVS
SDELIEGRGSDGNDLIQGGKGADFIEGGKGNDTIRDNSGHNTFLFSGHFGQDRIIGYQPTDRLVFQGADGSTDL
RDHAKAVGADTVLSFGADSVTLVGVGLGGLWSEGVLIS

> Fibroblast growth factor 1, LCD-based supercharged (FGF1(−))

MSRHHHHHHMFNLPPGNYQQPKLLYCSNGGHFLRILPDGTVDGTRDDSDQHIQLQLSAESVGEVYIKSTETGQY
LAMDTDGLLYGSQTPNEECLFLERLEENHYNTYISQEHAEQNWFVGLKQNGSCKDGPRTHYGQKAILFLPLPVS
SDELIEGRGSDGNDLIQGGKGADFIEGGKGNDTIRDNSGHNTFLFSGHFGQDRIIGYQPTDRLVFQGADGSTDL
RDHAKAVGADTVLSFGADSVTLVGVGLGGLWSEGVLIS

>SARS-CoV-2 spike protein N-terminal domain (NTD)

MSRHHHHHHVNLTTRTQLPPAYTNSFTRGVYYPDKVFRSSVLHSTQDLFLPFFSNVTWFHAIHVSGTNGTKRFD
NPVLPFNDGVYFASTEKSNIIRGWIFGTTLDSKTQSLLIVNNATNVVIKVCEFQFCNDPFLGVYYHKNNKSWME
SEFRVYSSANNCTFEYVSQPFLMDLEGKQGNFKNLREFVFKNIDGYFKIYSKHTPINLVRDLPQGFSALEPLVD
LPIGINITRFQTLLALHRSYLTPGDSSSGWTAGAAAYYVGYLQPRTFLLKYNENGTITDAVDCALDPLSETKCT
LKSELIEGRGSDGNDLIQGGKGADFIEGGKGNDTIRDNSGHNTFLFSGHFGQDRIIGYQPTDRLVFQGADGSTD
LRDHAKAVGADTVLSFGADSVTLVGVGLGGLWSEGVLIS

>SARS-CoV-2 spike protein N-terminal domain, LCD under +2 (NTD(LCD≤2))

MSRHHHHHHVNLTTRTQLPPAYTNSFTRGVYYPDKVFRSSVLHSTQDLFLPFFSNVTWFHAIHVSGTNGTDRFD
NPVLPFNDGVYFASTEKSNIIRGWIFGTTLDSKTQSLLIVNNATNVVIKVCEFQFCNDPFLGVYYHDNNESWME
SEFRVYSSANNCTFEYVSQPFLMDLEGKQGNFKNLREFVFENIDGYFKIYSDHTPINLVRDLPQGFSALEPLVD
LPIGINITRFQTLLALHRSYLTPGDSSSGWTAGAAAYYVGYLQPRTFLLEYNENGTITDAVDCALDPLSETKCT
LKSELIEGRGSDGNDLIQGGKGADFIEGGKGNDTIRDNSGHNTFLFSGHFGQDRIIGYQPTDRLVFQGADGSTD
LRDHAKAVGADTVLSFGADSVTLVGVGLGGLWSEGVLIS

>SARS-CoV-2 spike protein N-terminal domain, LCD under +1 (NTD(LCD≤1))
MSRHHHHHHVNLTTRTQLPPAYTNSFTDGVYYPDKVFDSSVLHSTQDLFLPFFSNVTWFHAIHVSGTNGTDRFD
NPVLPFNDGVYFASTEKSNIIRGWIFGTTLDSDTQSLLIVNNATNVVIKVCEFQFCNDPFLGVYYHKNNKSWME
SEFDVYSSANNCTFEYVSQPFLMDLEGKQGNFKNLREFVFDNIDGYFKIYSKHTPINLVDDLPQGFSALEPLVD
LPIGINITRFQTLLALHDSYLTPGDSSSGWTAGAAAYYVGYLQPRTFLLDYNENGTITDAVDCALDPLSETKCT
LKSELIEGRGSDGNDLIQGGKGADFIEGGKGNDTIRDNSGHNTFLFSGHFGQDRIIGYQPTDRLVFQGADGSTD
LRDHAKAVGADTVLSFGADSVTLVGVGLGGLWSEGVLIS

>SARS-CoV-2 spike protein receptor binding domain (RBD)
MSRHHHHHHPNITNLCPFGEVFNATRFASVYAWNRKRISNCVADYSVLYNSASFSTFKCYGVSPTKLNDLCFTN
VYADSFVIRGDEVRQIAPGQTGKIADYNYKLPDDFTGCVIAWNSNNLDSKVGGNYNYLYRLFRKSNLKPFERDI
STEIYQAGSTPCNGVEGFNCYFPLQSYGFQPTNGVGYQPYRVVVLSFELLHAPELIEGRGSDGNDLIQGGKGAD
FIEGGKGNDTIRDNSGHNTFLFSGHFGQDRIIGYQPTDRLVFQGADGSTDLRDHAKAVGADTVLSFGADSVTLV
GVGLGGLWSEGVLIS

>SARS-CoV-2 spike protein receptor binding domain, LCD under +2 (RBD(LCD≤2))
MSRHHHHHHPNITNLCPFGEVFNATRFASVYAWNRKDISNCVADYSVLYNSASFSTFKCYGVSPTKLNDLCFTN
VYADSFVIRGDEVRQIAPGQTGKIADYNYKLPDDFTGCVIAWNSNNLDSKVGGNYNYLYRLFRDSNLEPFERDI
STEIYQAGSTPCNGVEGFNCYFPLQSYGFQPTNGVGYQPYRVVVLSFELLHAPELIEGRGSDGNDLIQGGKGAD
FIEGGKGNDTIRDNSGHNTFLFSGHFGQDRIIGYQPTDRLVFQGADGSTDLRDHAKAVGADTVLSFGADSVTLV
GVGLGGLWSEGVLIS

>SARS-CoV-2 spike protein receptor binding domain, LCD under +1 (RBD(LCD≤1))
MSRHHHHHHPNITNLCPFGEVFNATRFASVYAWNRDDISNCVADYSVLYNSASFSTFKCYGVSPTDLNDLCFTN
VYADSFVIRGDEVRQIAPGQTGKIADYNYDLPDDFTGCVIAWNSNNLDSKVGGNYNYLYRLFDDSNLDPFERDI
STEIYQAGSTPCNGVEGFNCYFPLQSYGFQPTNGVGYQPYRVVVLSFELLHAPELIEGRGSDGNDLIQGGKGAD
FIEGGKGNDTIRDNSGHNTFLFSGHFGQDRIIGYQPTDRLVFQGADGSTDLRDHAKAVGADTVLSFGADSVTLV
GVGLGGLWSEGVLIS

>pDART Translation Structure [14]
MSRHMGTASELIEGRGSDGNDLIQGGKGADFIEGGKGNDTIRDNSGHNTFLFSGHFGQDRIIGYQPTDRLVFQG
ADGSTDLRDHAKAVGADTVLSFGADSVTLVGVGLGGLWSEGVLIS

>pBR05 Translation Structure
MSRHMGTASELRRRRRGIEGRGSDGNDLIQGGKGADFIEGGKGNDTIRDNSGHNTFLFSGHFGQDRIIGYQPTD
RLVFQGADGSTDLRDHAKAVGADTVLSFGADSVTLVGVGLGGLWSEGVLIS

>pBR10 Translation Structure [14]
MSRHMGTASELRRRRRRRRRRGIEGRGSDGNDLIQGGKGADFIEGGKGNDTIRDNSGHNTFLFSGHFGQDRIIG
YQPTDRLVFQGADGSTDLRDHAKAVGADTVLSFGADSVTLVGVGLGGLWSEGVLIS

>pBK05 Translation Structure
MSRHMGTASELKKKKKGIEGRGSDGNDLIQGGKGADFIEGGKGNDTIRDNSGHNTFLFSGHFGQDRIIGYQPTD

RLVFQGADGSTDLRDHAKAVGADTVLSFGADSVTLVGVGLGGLWSEGVLIS

>pBK10 Translation Structure
MSRHMGTASELKKKKKKKKKKGIEGRGSDGNDLIQGGKGADFIEGGKGNDTIRDNSGHNTFLFSGHFGQDRIIG
YQPTDRLVFQGADGSTDLRDHAKAVGADTVLSFGADSVTLVGVGLGGLWSEGVLIS

>pBRD10 Translation Structure
MSRHMGTASELRDRDRDRDRDRDRDRDRDRDGIEGRGSDGNDLIQGGKGADFIEGGKGNDTIRDNSGHNTFLFS
GHFGQDRIIGYQPTDRLVFQGADGSTDLRDHAKAVGADTVLSFGADSVTLVGVGLGGLWSEGVLIS

>pBRN10 Translation Structure
MSRHMGTASELRNRNRNRNRNRNRNRNRNRNGIEGRGSDGNDLIQGGKGADFIEGGKGNDTIRDNSGHNTFLFS
GHFGQDRIIGYQPTDRLVFQGADGSTDLRDHAKAVGADTVLSFGADSVTLVGVGLGGLWSEGVLIS

>pBKD10 Translation Structure
MSRHMGTASELKDKDKDKDKDKDKDKDKDKDGIEGRGSDGNDLIQGGKGADFIEGGKGNDTIRDNSGHNTFLFS
GHFGQDRIIGYQPTDRLVFQGADGSTDLRDHAKAVGADTVLSFGADSVTLVGVGLGGLWSEGVLIS

>pBKN10 Translation Structure
MSRHMGTASELKNKNKNKNKNKNKNKNKNKNGIEGRGSDGNDLIQGGKGADFIEGGKGNDTIRDNSGHNTFLFS
GHFGQDRIIGYQPTDRLVFQGADGSTDLRDHAKAVGADTVLSFGADSVTLVGVGLGGLWSEGVLIS

Table S1. Primers used to construct the plasmids for the positively charged patch experiments.

Name	Sequence	Features
F-Arg10	TGGGTACCGCTAGC **GAGCTC** CGTCGCCGACGGC GTCGCCGACGGCGTCGC	Complementary region, SacI site, insert sequence
R-Arg10	CCTCGTCCTTCAATG**CC** GCGACGCCGTCGGCGAC GCCGTCGGCGACG	Complementary region, frame-keeper, insert sequence
F-Arg05	TGGGTACCGCTAGC **GAGCTC** CGTCGCCGACGGC GT	Complementary region, SacI site, insert sequence
R-Arg05	CCTCGTCCTTCAATG**CC** ACGCCGTCGGCGACG	Complementary region, frame-keeper, insert sequence
F-Lys10	TGGGTACCGCTAGC **GAGCTC** AAGAAAAAGAAA AAGAAGAAAAAGAAAAAG	Complementary region, SacI site, insert sequence
R-Lys10	CCTCGTCCTTCAATG**CC** CTTTTTCTTTTTCTTCTTTT TCTTTTTCTT	Complementary region, frame-keeper, insert sequence
F-Lys05	TGGGTACCGCTAGC **GAGCTC** AAGAAAAAGAAA AAG	Complementary region, SacI site, insert sequence
R-Lys05	CCTCGTCCTTCAATG**CC** CTTTTTCTTTTTCTT	Complementary region, frame-keeper, insert sequence
F-ArgAsp10	TGGGTACCGCTAGC **GAGCTC** CGTGATCGCGACC GAGATCGGGATCGTGACCGCGATCGAGAT	Complementary region, SacI site, insert sequence
R-ArgAsp10	CCTCGTCCTTCAATG**CC** GTCCCGGTCACGGTCCC GATCTCGATCGCGGTCACGATCCCG	Complementary region, frame-keeper, insert sequence
F-ArgAsn10	TGGGTACCGCTAGC **GAGCTC** CGTAATCGCAACC GAAATCGGAATCGTAACCGCAATCGAAAT	Complementary region, SacI site, insert sequence
R-ArgAsn10	CCTCGTCCTTCAATG**CC** GTTCCGGTTACGGTTCCG ATTTCGATTGCGGTTACGATTCCG	Complementary region, frame-keeper, insert sequence
F-LysAsp10	TGGGTACCGCTAGC **GAGCTC** AAGGATAAGGAC AAAGATAAAGACAAGGACAAAGACAAAGAT	Complementary region, SacI site, insert sequence
R-LysAsp10	CCTCGTCCTTCAATG**CC** ATCCTTGTCTTTATCCTT ATCTTTGTCTTTGTCCTTGTCTTT	Complementary region, frame-keeper, insert sequence
F-LysAsn10	TGGGTACCGCTAGC **GAGCTC** AAGAATAAGAAC AAAAATAAAAACAAGAACAAAAACAAAAAT	Complementary region, SacI site, insert sequence
R-LysAsn10	CCTCGTCCTTCAATG**CC** ATTTTTGTTTTTGTTCTTG TTTTTATTTTTGTTCTTATTCTT	Complementary region, frame-keeper, insert sequence
F-LysAsp03	TGGGTACCGCTAGC **GAGCTC** AAGGATAAAGAC AAG **GA**	Complementary region, SacI site, insert sequence
R-LysAsp03	TTGTCCTTATCCTTG**TC** CTTGTCTTTATCCTT **GAG CT**	Complementary region, frame-keeper, insert sequence
F-LysAsn03	TGGGTACCGCTAGC **GAGCTC** AAGAATAAAAAT AAA **AA**	Complementary region, SacI site, insert sequence
R-LysAsn03	TTGTTCTTATTCTTG**TT** TTTATTTTTATTCTT **GAGC T**	Complementary region, frame-keeper, insert sequence

Table S2. List of genes studied in this experiment.

Abbrev.	Full name	Source	Source type
TliA	Thermostable lipase A	*Pseudomonas fluorescens* SIK-W1	Genomic DNA
NKC-TliA	NKC-TliA	[14, 42]	Constructed
CTP-TliA	CTP-TliA	[14, 43]	Constructed
GST	Glutathione S-transferase	*Sus scrofa domesticus*	Plasmid
GST(−20)	GST, −20 negatively supercharged	[15]	Synthesized
GST(+19)	GST, +19 positively supercharged	Manually supercharged	Synthesized
SAv	Streptavidin	*Streptomyces avidinii*	Plasmid
SAv(−10)	SAv, −10 negatively supercharged	[15]	Synthesized
SAv(+13)	SAv, +13 positively supercharged	[15]	Synthesized
Cuti	Cutinase	*Nectria haematococca*, [14]	Plasmid
Cuti(−)	Cutinase, negatively supercharged	AvNAPSA supercharging, threshold = 100	Synthesized
Chi	Chitinase	*Bacillus thuringenesis*, [14]	Plasmid
Chi(−)	Chitinase, negatively supercharged	AvNAPSA supercharging, threshold = 100	Synthesized
MelC2	MelC2 Tyrosinase	*Streptomyces antibioticus*	Plasmid
MelC2(−40)	MelC2, −40 negatively supercharged	AvNAPSA supercharging, threshold = 120	Synthesized
MelC2(−20)	MelC2, −20 negatively supercharged	AvNAPSA supercharging, threshold = 100	Synthesized
MelC2(Q)	MelC2, selectively superneutralized	Manually superneutralized	Synthesized
M37	M37 lipase	*Photobacterium lipolyticum*, [14]	Genomic DNA
M37(−23)	M37, −23 negatively supercharged	AvNAPSA supercharging, threshold = 100	Synthesized
M37(−14)	M37 lipase, −14 negatively supercharged	AvNAPSA supercharging, threshold = 90	Synthesized
M37(Q)	M37, selectively superneutralized	Manually superneutralized	Synthesized
M37(var)	M37, randomly mutated and screened	Random mutation and activity screening	Synthesized via mixed-base
TGFβ	Transforming growth factor beta	*Homo sapiens* [44]	Synthesized
TGFβ(−)	TGFβ, LCD-based negatively supercharged	Linear charge density-based supercharging	Synthesized
TNFβ	Tumor necrosis factor beta	*Homo sapiens* [45]	Synthesized
TNFβ(−)	TNFβ, LCD-based negatively supercharged	Linear charge density-based supercharging	Synthesized
FGF1	Fibroblast growth factor beta	*Homo sapiens* [46]	Synthesized
FGF1(−)	FGF1, LCD-based negatively supercharged	Linear charge density-based supercharging	Synthesized
